# A Clinical Conundrum in Patients With a Common Clinical Spectrum but Rare Differentials: Hyper-Immunoglobulin E Syndrome and Idiopathic Eosinophilia

**DOI:** 10.7759/cureus.89898

**Published:** 2025-08-12

**Authors:** Somnath Bhattacharya, Prashant Yadav, Adesh Kumar

**Affiliations:** 1 Department of Respiratory Medicine, Uttar Pradesh University of Medical Sciences, Etawah, IND

**Keywords:** erythematous rash, hyper immunoglobulin e syndrome (hies), idiopathic eosinophilia, idiopathic hyper eosinophilic syndrome(ihes), serum ige, sinopulmonary infection

## Abstract

Hyper-immunoglobulin E syndrome (HIES) and idiopathic eosinophilia lie on the same spectrum of immunodeficiency disease; therefore, physicians need to consider both possibilities as differential diagnoses for early identification. HIES is a primary immunodeficiency syndrome characterized by markedly elevated total IgE levels along with recurrent eczematous rash, skin infections, sinopulmonary infections, and multiple hospitalizations. Similarly, patients with idiopathic hypereosinophilic syndrome (HES) also present with marked eosinophilia, recurrent sinopulmonary infections, and evidence of organ damage such as eosinophilic myositis. Idiopathic eosinophilia also lacks any identifiable secondary aetiology and evidence of organ damage, with only eosinophilia in bone marrow. Both our cases have similar symptoms, presenting in the busy outpatient department settings that needed thoughtful consideration for the differentials. Awareness among Clinicians about this wide spectrum of differentials with overlapping presentations will facilitate early diagnosis and eventually enable optimal cost-effective treatment for patients.

## Introduction

Day-to-day clinical practice requires a high degree of clinical suspicion and knowledge of rare entities; very often, they present with common manifestations and symptoms that are often overlooked. Hyper‑immunoglobulin E syndrome (HIES) is a rare, multisystem primary immunodeficiency disorder, occurring in one in a million individuals, characterized by recurrent eczematoid rash, sinopulmonary and skin infections, with markedly elevated isolated serum‑immunoglobulin (Ig)E levels [1]. The familial form is uncommon and classified as Type‑1 HIES (Job syndrome), which is autosomal dominant (AD‑HIES). It is characterised by immune system abnormalities associated with skeletal, connective, and vascular tissues [2,3]. Whereas Type‑2 HIES, being autosomal recessive (AR‑HIES), involves more of recurrent skin and sinopulmonary infections but lacks musculoskeletal manifestations [2-4]. AD-HIES occurs due to a mutation in the human signal transducer and activator of transcription 3 gene (STAT3) [5]. On the other hand, AR-HIES occurs due to DOCK8 gene mutations, but most clinical cases are sporadic in nature [5]. Similar clinical manifestations, but with organ and tissue damage, may also be seen with isolated hyper eosinophilia. To address this issue, WHO endorsed the empirical diagnostic criteria for hypereosinophilic syndrome in 2016, which includes A) blood peripheral eosinophil count >1500/mm^3^ for more than six months, B) evidence of organ, such as heart, dysfunction or damage and C) absence of identifiable causes for eosinophilia and eosinophilic blasts in the peripheral blood [6-10]. Confirmatory diagnosis of Idiopathic HES (IHES) can thus be made by exclusion of possible etiologies and fulfilment of all three criteria [11]. But the diagnosis of idiopathic eosinophilia can only be made in the absence of organ damage and presence of idiopathic hyper eosinophilia. This broad spectrum of diagnosis, from sporadic HIES to Idiopathic eosinophilia, often presents with similar symptoms, as seen in our two cases. This underscores the importance of increasing awareness among clinicians to enable early recognition and appropriate management.

## Case presentation

Case 1

**Figure 1:**
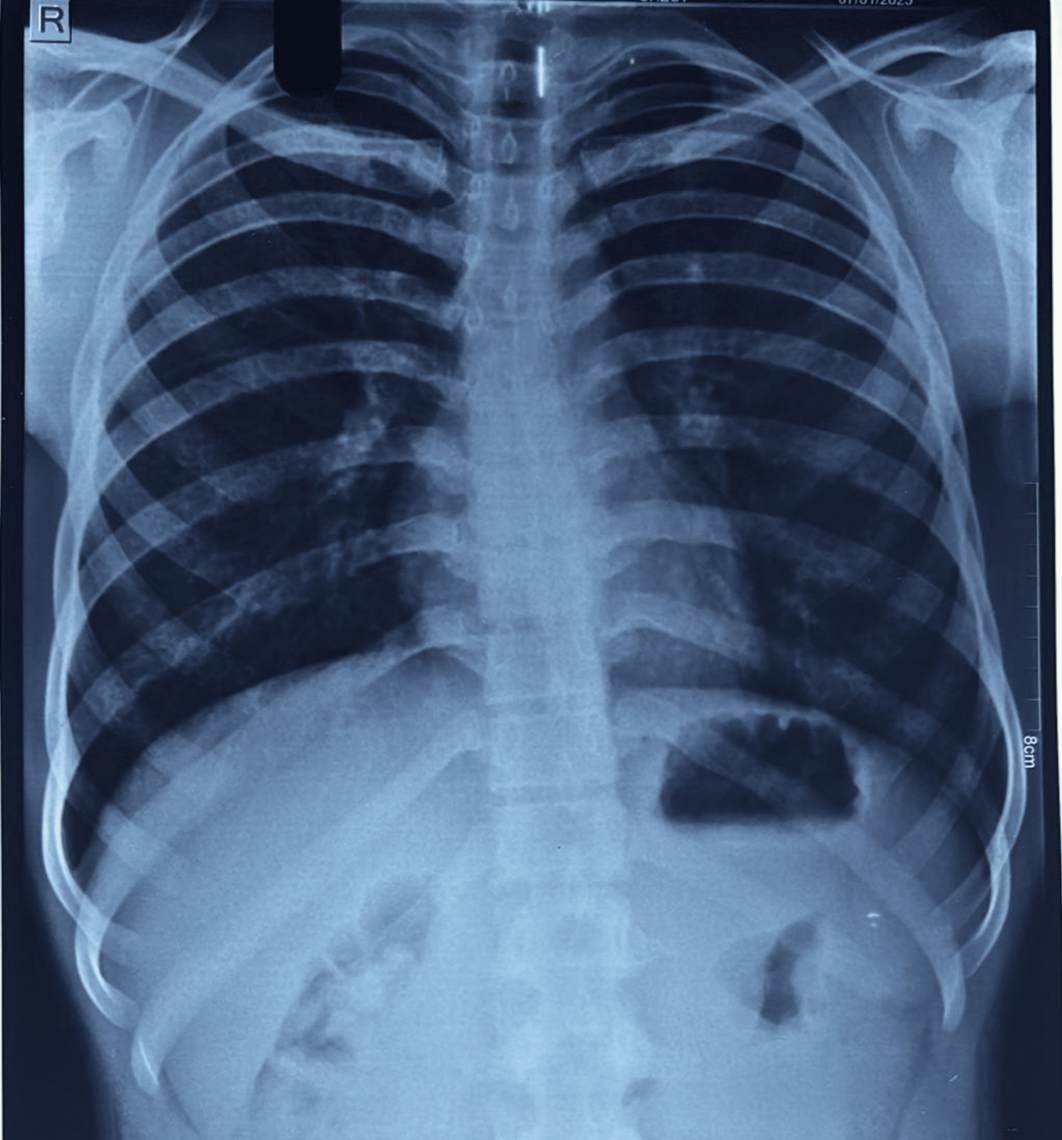
Chest X-ray PA view in Case 1 Normal chest X-ray of the Case 1 patient. PA: Postero-anterior.

A 28-year-old male presented to the outpatient department with complaints of recurrent rhino sinusitis, persistent cough, and chest tightness for the last eight months. He also suffered from recurrent chest infections and erythematous rashes for the past two years. No history of similar illness in family or any symptoms of other organ involvement and drug abuse. Routine blood investigations showed a very high serum IgE 2966 IU/ml (<3781 U/ml) level with normal IgG 1364 mg/dl (650-1600mg/dl), IgA 233.70 mg/dl (40-350mg/dl), and IgM 176.10 mg/dl (50-300 mg/dl). Serum reports of Aspergillus-specific IgE 0.24 kUA/L (<0.35 kUA/L) and IgG 25.4mg/dl (<27 mg/dl) were negative. Stool examination for ova and parasites was negative, and the skin prick test was found positive for house dust mite only. Chest X-ray and HRCT thorax did not show any obvious abnormalities (Figure 1). Post bronchodilator FEV1/FVC ratio >0.7, FEV1> 80% predicted, FEF25-75> 80% predicted, with no bronchodilator reversibility seen in Spirometry testing. Routine haematological examination did not show any cellular abnormality with an absolute eosinophil count of 100 cells/cm^3^. He was treated with oral antihistamines, intravenous antibiotics, deworming medicines, and inhalers as needed. Later, a follow-up investigation showed STAT3 mutation to be negative. However, repeated estimation of total IgE after one month remained in the range of >3000 IU/ml, and the diagnosis of HIES (Sporadic) was made.

Case 2

**Figure 2:**
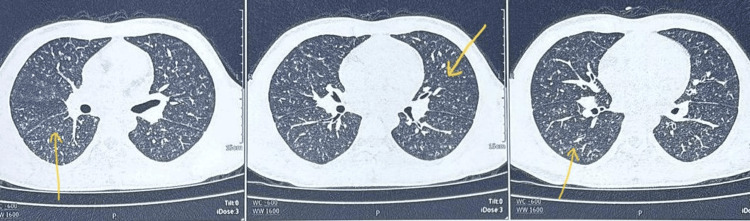
HRCT thorax in Case 2 showing diffuse centrilobular nodules HRCT: High-Resolution Computed Tomography The yellow arrows are showing bilateral diffuse centrilobular nodules.

**Figure 3:**
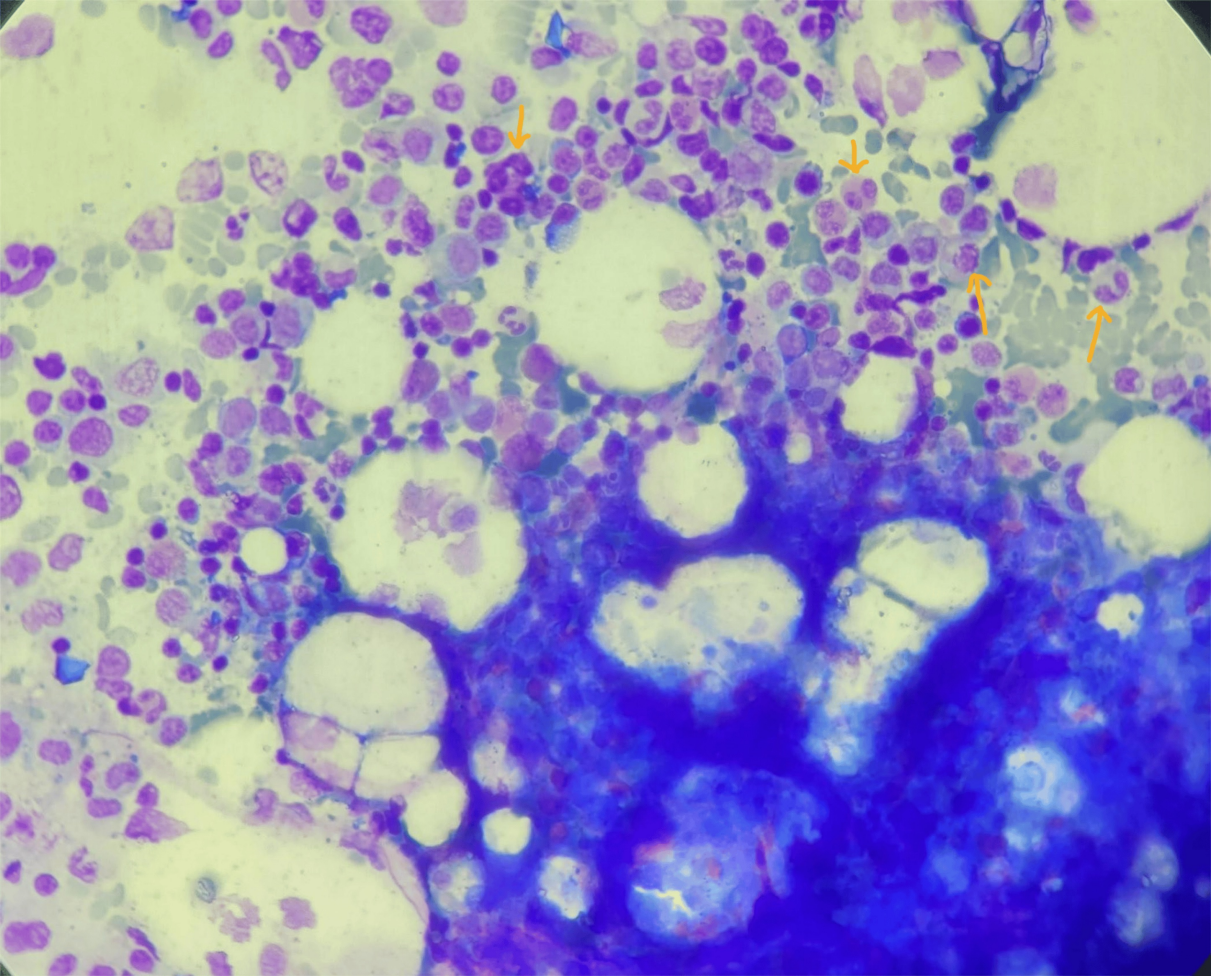
Bone marrow examination after Giemsa stain in Case 2 The arrows are showing an abundance of eosinophils and their precursors (35%).

A 17-year-old male visited the outpatient department with complaints of recurrent rhinitis, dry cough, and fever on and off for the last six months. He was suffering from recurrent chest infections and rhinosinusitis over the last three to four years. There was no history of similar illness in the family or any symptoms of other organ involvement or drug abuse. Chest X-ray appears to be normal, but high-resolution computed tomography (HRCT) of the thorax shows bilateral diffuse centrilobular nodules pointed by the arrow in Figure 2. Routine blood investigations showed a high count of total leucocytes (28,700 cells/cm^3^), CRP 2.4mg/L with eosinophils 79.7%, neutrophils 4.4%, and lymphocytes 12.8%. Total IgE was 142.01 U/ml, Aspergillus-specific IgE 0.31 kUA/L (<0.9 kUA/L), and antifilarial antigens were nonreactive. Serology, antineutrophil cytoplasmic antibodies (ANCA), and HIV status were nonreactive too. His stool sample was found to be negative for ova and parasites. His blood test revealed low serum LDH, and a low Vitamin B12 level of 180 pg/ml (200-900 pg/ml). ECG and echocardiogram showed no abnormalities with ejection fraction (EF) 60%. He also underwent bronchoscopy-guided bronchoalveolar lavage (BAL) examination with eosinophils 1% and BAL GeneXpert being negative. Blood culture and urine culture results were negative. He was treated symptomatically and with antibiotics like diethylcarbamazine for two weeks. On follow-up, he remained stable with minimal symptoms, but total leucocyte count was 21700 cells/cm^3^, eosinophils 75.4%, and absolute eosinophil count was 20300 cells/cm^3. ^Following these, he was sent for bone marrow examination. The reports of bone marrow revealed myeloid to erythroid (M:E) ratio 11:1, eosinophil and precursors (myelocytes, metamyelocytes, band) 35%, myeloblast 1% suggesting the diagnosis of idiopathic eosinophilia as shown in (Figure 3) by pointers.

## Discussion

As described in the literature, HIES is a rare primary immunodeficiency syndrome with the majority of cases occurring sporadically [5]. AD‑HIES shows a STAT3 mutation, whereas AR‑HIES commonly involves DOCK8 gene mutations [5]. In 1966, Davis, Wedgwood, and Schaller coined the name “Job’s syndrome,” attributing the clinical symptoms to the affliction suffered by Prophet Job [12]. In 1972, Buckley et al. further described and elaborated the clinical description, which was also called Buckley’s syndrome [12]. Clinically, most patients present with recurrent eczematous rashes and sinopulmonary infections with extremely high total IgE > 2000 IU with eosinophilia. Hence, these findings are very similar to the first case demonstrated. Our patient was clinically stable with very high persistent total IgE > 3000 IU with recurrent erythematous rashes and sinopulmonary infections, but no eosinophilia over the years that were controlled with medications like montelukast, anti-histaminics, and antibiotics. Multisystem involvement, like skeletal, vascular, or connective tissue, is a very rare presentation, which was also absent in our case. Clinically, the patient’s features favoured AR/sporadic nature in view of recurrent skin lesions and lung infections. Later, follow-up from outside reports showed STAT3 mutation was negative. 

The second case also presented with similar clinical symptoms in the outpatient department, but with very high eosinophilia and rather normal total IgE levels. Secondary causes of hyper eosinophilia, like parasitic infection, filariasis, ABPA, EGPA, etc., were ruled out. To establish the diagnosis of Idiopathic eosinophilia, bone marrow examination findings and lack of evidence of organ damage, such as endomyocardial fibrosis or eosinophilic myositis in this patient, were conclusive. In 2016, the WHO reaffirmed the diagnostic criteria for hypereosinophilic syndrome (HES), as previously outlined, and emphasized the consideration of clonal mutations associated with myeloid/lymphoid neoplasms with eosinophilia. This includes gene rearrangements involving FIP1L1-PDGFRA, PDGFRB, FGFR1, or PCM1-JAK2 [11,12]. Lack of evidence of any organ damage, stable clinical condition with low Vitamin B12 levels, low serum LDH, and bone marrow examination with 35% eosinophils with precursors and no blast cells favoured the diagnosis of idiopathic eosinophilia more than clonal proliferation. Recently, drugs like imatinib have shown some good response in patients with clonal mutations like FIP1L1-PDGFRA [11-13]. Likewise, the role of dupilumab in HIES, particularly with atopic dermatitis, has shown promise [13].

## Conclusions

Rare diseases often present with common symptoms and manifestations in our day-to-day busy outpatient clinics. Clinicians need to stay alert, monitor patients closely, and be aware of rare conditions to ensure early and accurate diagnosis. Genetic mutation analyses are now becoming an integral part of the diagnostic and therapeutic armamentarium. However, the availability and cost-benefit ratio is still a matter of concern in resource-limited settings.
